# Exploring the Integrated Role of miRNAs and lncRNAs in Regulating the Transcriptional Response to Amino Acids and Insulin-like Growth Factor 1 in Gilthead Sea Bream (*Sparus aurata*) Myoblasts

**DOI:** 10.3390/ijms25073894

**Published:** 2024-03-31

**Authors:** Isabel García-Pérez, Bruno Oliveira Silva Duran, Maeli Dal-Pai-Silva, Daniel Garcia de la serrana

**Affiliations:** 1 Department of Cell Biology, Physiology and Immunology, Faculty of Biology, University of Barcelona (UB), 08028 Barcelona, Spain; isabelgarcia@ub.edu; 2 Department of Histology, Embryology and Cell Biology, Institute of Biological Sciences, Federal University of Goiás (UFG), Goiânia 74690-900, Brazil; brunoduran@ufg.br; 3 Department of Structural and Functional Biology, Institute of Biosciences, São Paulo State University (UNESP), Botucatu 18618-689, Brazil; maeli.dal-pai@unesp.br

**Keywords:** microRNAome, transcriptome, omics, RNA interactions, bioinformatics, fish, muscle growth, amino acids, Igf-1

## Abstract

In this study, gilthead sea bream (*Sparus aurata*) fast muscle myoblasts were stimulated with two pro-growth treatments, amino acids (AA) and insulin-like growth factor 1 (Igf-1), to analyze the transcriptional response of mRNAs, microRNAs (miRNAs) and long non-coding RNAs (lncRNAs) and to explore their possible regulatory network using bioinformatic approaches. AA had a higher impact on transcription (1795 mRNAs changed) compared to Igf-1 (385 mRNAs changed). Both treatments stimulated the transcription of mRNAs related to muscle differentiation (GO:0042692) and sarcomere (GO:0030017), while AA strongly stimulated DNA replication and cell division (GO:0007049). Both pro-growth treatments altered the transcription of over 100 miRNAs, including muscle-specific miRNAs (myomiRs), such as *miR-133a/b*, *miR-206*, *miR-499*, *miR-1*, and *miR-27a*. Among 111 detected lncRNAs (>1 FPKM), only 30 were significantly changed by AA and 11 by Igf-1. Eight lncRNAs exhibited strong negative correlations with several mRNAs, suggesting a possible regulation, while 30 lncRNAs showed strong correlations and interactions with several miRNAs, suggesting a role as sponges. This work is the first step in the identification of the ncRNAs network controlling muscle development and growth in gilthead sea bream, pointing out potential regulatory mechanisms in response to pro-growth signals.

## 1. Introduction

The skeletal muscle of teleost fish is a very plastic tissue that integrates external and internal inputs to adapt to a changing environment. Most teleost can form new muscle fibers (hyperplasia) long after the end of metamorphosis well into adulthood (until about 44% of the maximal length of the species), overlapping with the growth of pre-existent fibers (hypertrophy) [1,2,3]. Muscle growth includes the activation of the satellite cells, their proliferation, fusion, differentiation, and maturation in a process known as myogenesis. The molecular regulation of myogenesis involves the coordinated work of transcription factors, growth factors, activation of different pathways, and fusion proteins [2,4,5,6,7,8].

Furthermore, muscle growth and development also depend on the balance between protein synthesis and degradation, which are controlled by various cellular signaling pathways, including the nutrient-sensitive mechanistic target of rapamycin (mTOR) network. mTOR is a protein kinase that acts as a central regulator of cellular metabolism, proliferation, and growth and is activated in response to various signals, including nutrients (e.g., amino acids (AA)) and growth factors (e.g., insulin-like growth factor 1 (Igf-1)). When AA are present in sufficient quantities, they activate mTOR at the lysosome membrane [9,10,11,12], which then stimulates protein synthesis by phosphorylating and activating downstream targets such as P70 ribosomal S6 kinase (P70S6K) and eukaryotic translation initiation factor 4e binding protein 1 (4EBP1). Studies with fish muscle showed that AA reduce transcription of muscle-specific ubiquitin ligases [13] and autophagy-related genes [14], leading to reduced protein breakdown [15,16]. Likewise, Igf-1 also activates mTOR throughout the induction of phosphatidylinositol 3-kinase (PI3K)/protein kinase B (AKT) pathway. It has been shown in different fish species and experimental setups that Igfs increase muscle growth by promoting myoblast proliferation and differentiation [17,18,19,20]. Thus, given the importance of the Igf system and AA in promoting protein synthesis and in the myogenesis process, studying their effects would help to better understand the potential regulation of muscle growth and development in fish, including aquaculture species like the gilthead sea bream (*Sparus aurata*).

In the last decades, research in mammalian models has demonstrated that the non-coding RNAs (ncRNAs) also play a key role in regulating myogenesis [21,22,23], but little is known about their role in fish muscle development [24,25,26,27]. The ncRNAs are a group of RNAs that, generally, do not codify for proteins but perform various regulatory functions in cellular processes. The ncRNAs include ribosomal RNAs (rRNAs), transfer RNAs (tRNAs), small nuclear RNAs (snRNAs), small nucleolar RNAs (snoRNA), microRNAs (miRNAs), small interfering RNA (siRNAs), piwi-interacting RNAs (piRNAs), circular RNAs (circRNAs) and long non-coding RNAs (lncRNAs). The miRNAs regulate gene expression by recognition of the complementary sequence present in the target mRNAs. When an mRNA is recognized by a specific miRNA, its degeneration, deadenylation, or impaired translation into a protein can be triggered, usually leading to a negative correlation between the expression of miRNAs and their target mRNAs [28,29,30]. Therefore, the miRNAs expand the complexity of transcriptomic regulation and are key players in the control of cellular functions [31]. Many miRNAs are ubiquitously expressed in most cell types and tissues, but some are tissue-specific like the myomiRs, muscle enriched, or striated muscle-specific miRNAs. MyomiRs are involved in myoblast proliferation, differentiation, or muscle regeneration, and each one is expressed differently throughout the myogenesis process [22,32,33]. It has been shown in C2C12 myoblasts that *miR-1* and *miR-206* promote myogenic differentiation by repressing the expression of Pax7 and histone deacetylase 4 (HDAC4) [34,35,36,37]; and also that *miR-206* is involved in muscle regeneration and it is markedly upregulated in satellite cells following muscle injury [38,39]. On the other hand, *miR-133a* is known to have an important role in muscle cell proliferation, repressing serum response factor (SRF) [40]. However, *miR-133b* also participates in the promotion of myoblast differentiation and fusion [41,42]. In the case of *miR-499* and *miR-208b*, they are involved in the specification and maintenance of slow-twitch phenotype [43,44,45]. The roles of these miRNAs were also investigated in fish skeletal muscle, with *miR-1/206* and *miR-133* families regulating myogenesis and development [46,47,48], sarcomeric organization [49], and protein balance [50,51,52]; and *miR-499* inducing the establishment and maintenance of slow-twitch muscle fibers [48,53,54].

On the other hand, lncRNAs can increase or decrease the transcription and function of genes through different strategies, such as direct interaction with the DNA, RNA, or even proteins. Some lncRNAs can interact with the DNA and change the chromatin structure, modulating the access of transcription factors to the gene promotors or allowing the physical proximity to enhancers [55,56,57]. In addition, the lncRNAs can also interact directly with mRNAs, showing opposite functions that could induce mRNA degradation or prevent it by acting as sponges of miRNAs [26,58], or even regulate the gene function by direct interaction with proteins [59]. The number of lncRNAs characterized in human skeletal muscle has increased in recent years and now includes lncRNAs such as *Neat1* [60], *MUNC* [61,62], *linc-RAM* [63], *Irm* [64], or *H19* [65]. Recent research in fish has indicated that lncRNAs participate in many biological processes, including lipid metabolism [66], intestinal homeostasis [67], immune response [68], sex differentiation [69], and the smoltification process [70]. However, our knowledge of lncRNAs in fish skeletal muscle is very limited [24,26,27,71]. One of the major problems is the apparent low conservation of lncRNAs [72], which makes it very difficult to identify relevant lncRNAs in species other than humans, having to start from scratch the work in different species.

To our knowledge, the role of miRNAs and lncRNAs in regulating the transcriptional response of fish skeletal muscle to pro-growth signals such as AA and Igf-1 has not yet been investigated in fish. Hence, this work uses an RNA-Seq approach to address the present lack of knowledge by generating a transcriptome and microRNAome from gilthead sea bream myoblasts stimulated with AA or Igf-1, and study the interactions between mRNAs, miRNAs, and lncRNAs to better understand the role of ncRNAs in the myoblast’s transcriptional response to pro-growth signals.

## 2. Results

### 2.1. Identification of miRNAs and lncRNAs in Gilthead Sea Bream Myoblasts

**Figure 1:**
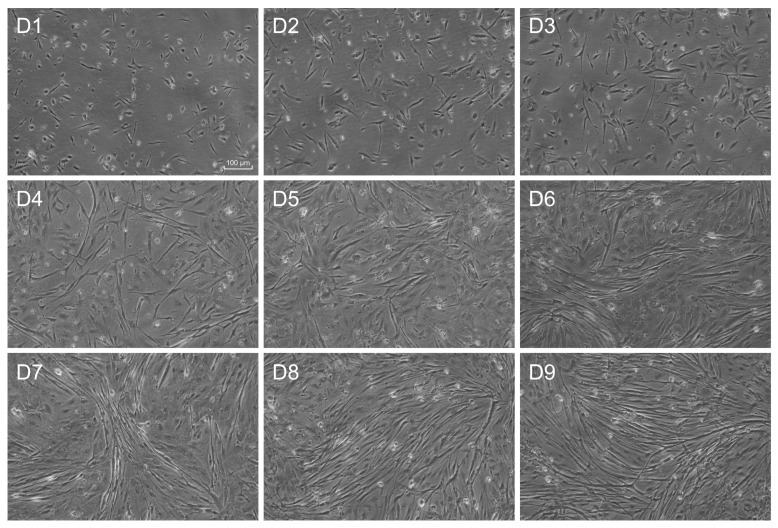
Representative bright-field images of muscle cells throughout the culture, from day 1 to day 9.

**Figure 2:**
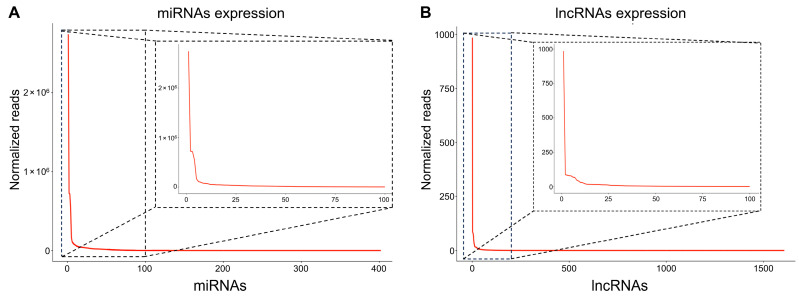
miRNAs (**A**) and lncRNAs (**B**) transcription levels identified in 8 days developed gilthead sea bream myoblasts. Transcription levels of lncRNAs are expressed as FPKM (fragments per kilobase of exon per million fragments mapped), while miRNAs are expressed as normalized reads. The insert represents the expression of the first 100 miRNAs and lncRNAs.

Myoblasts from gilthead sea bream fast skeletal muscle were extracted and seeded on 6-well culture plates at a density of 1.5 × 10^6^ cells/well and let to develop for 8 days (Figure 1). At day 8, myoblasts were still proliferating, but a significant proportion of them started to fuse and form myotubes, allowing us to investigate miRNAs and lncRNAs present during proliferation and differentiation in response to pro-growth signals. We detected 403 miRNAs expressed in the gilthead sea bream myoblasts, with 8.58% showing a high expression (over 10,000 normalized reads), 20% showing low expression (under 10 normalized reads), and 70% showing intermediate expression (between 10 and 10,000 normalized reads) (Figure 2A). We also identified more than 870 lncRNAs (over 0.001 FPKM), but only 111 had over 1 FPKM average expression, while 25 had over 10 FPKM (Figure 2B). It is interesting to notice that in both lncRNAs and miRNAs the transcriptomic landscape is dominated by a few of them (Figure 2). For instance, four miRNAs (*miR-21*, *miR-146*, *miR-22*, and *miR-206*) were found to have over 500,000 normalized reads (Figure 2A; Supplementary File S1). Other miRNAs known to be important in mammalian skeletal muscle (*miR-26a*, *miR-27*, *miR-133a/b*, *miR-221/222*, *miR-1*, or *miR-499*) were also relatively abundant but not at the same level (Figure 2A; Supplementary File S1). In the case of lncRNAs, one of them, *ENSSAUG00010015132*, showed ten times more expression (>900 average FPKM) than the second more expressed lncRNA (*ENSSAUG00010022378*; >80 average FPKM), which rapidly decreased to very low levels of expression for the others lncRNAs (Figure 2B; Supplementary File S1). The majority of lncRNAs were predicted to be either located in the cytoplasm (70%) or nucleus (28%) (Supplementary File S1). A BLAST search of the lncRNAs > 1 FPKM from gilthead sea bream against the human and mouse genome did not show any significant ortholog.

### 2.2. Transcriptomic Changes of mRNAs in Response to AA and Igf-1

**Figure 3:**
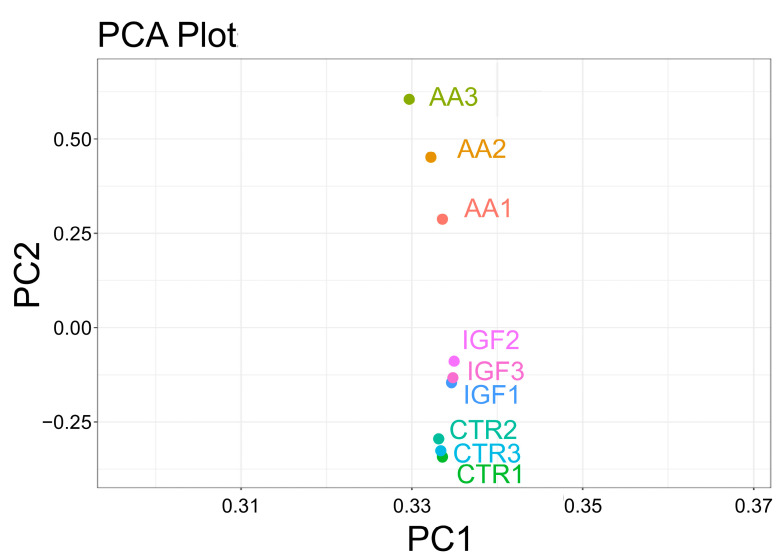
Principal component analysis (PCA) plot showing gene expression data grouped according to the CTR, AA, and Igf-1 treatments.

To determine the effects of the treatments, a principal components analysis (PCA) was performed. The PCA analysis showed that the samples from each condition clustered together in three distinct groups. It is interesting to notice that the Igf-1 cluster was closer to the CTR cluster than the AA cluster, suggesting that the global transcriptomic profile of the myoblasts treated with Igf-1 was more similar to the CTR profile than to that of AA (Figure 3). Also, the replicates of the Igf-1 and CTR groups were closer to each other compared to the AA groups, indicating lower variability in the response to the treatments (Figure 3).

**Figure 4:**
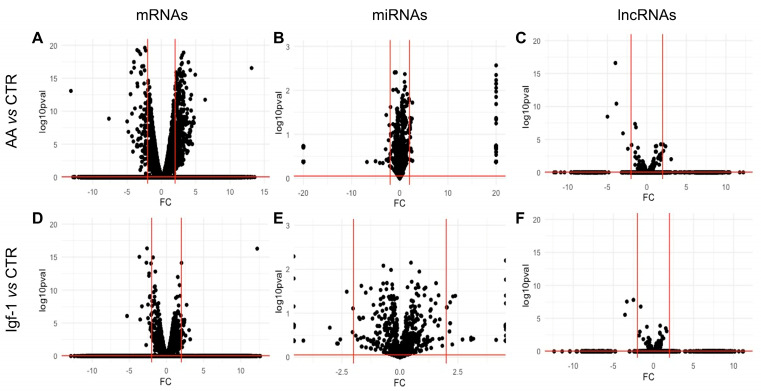
Volcano plots of differentially expressed mRNAs, lncRNAs, and miRNAs detected in gilthead sea bream myoblasts in response to the treatments. Volcano plots of transcription results of the AA vs. CTR group (**A**–**C**) and Igf-1 vs. CTR group (**D**–**F**) for mRNAs (**A**,**D**), miRNAs (**B**,**E**), and lncRNAs (**C**,**F**). Red vertical lines represent log2FC of 1 and −1. Red horizontal lines represent a *p*-value of 0.05.

**Figure 5:**
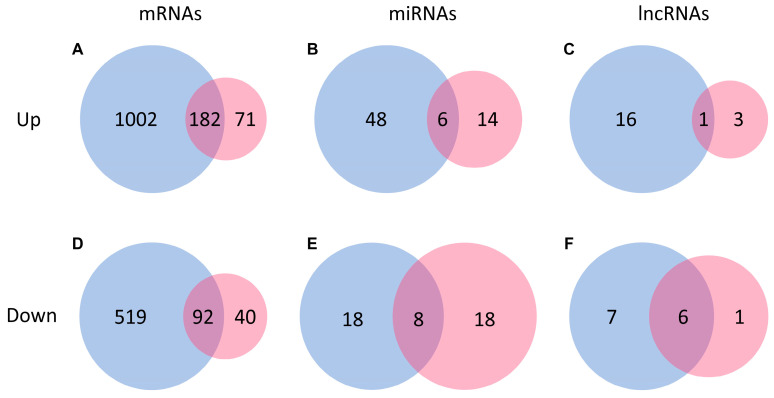
Venn diagrams of mRNAs, miRNAs, and lncRNAs significantly modified by AA and Igf-1. Venn diagrams showing the number of mRNAs, miRNAs, and lncRNAs upregulated (**A**–**C**) and downregulated (**D**–**F**) in response to the treatments. The numbers inside the blue bubbles and red bubbles represent the number of mRNAs (**A**,**D**), miRNAs (**B**,**E**), and lncRNAs (**C**,**F**) uniquely changed in response to AA and Igf-1, respectively. The number in the intersection of the two bubbles indicates the mRNAs, miRNAs, and lncRNAs that commonly changed in response to both treatments.

The transcriptional response of the gilthead sea bream myoblasts to AA was more intense than the response to only Igf-1 (Supplementary File S2). In response to AA, we found a total of 1184 upregulated and 611 downregulated mRNAs compared to the CTR myoblasts (Figure 4 and Figure 5). When Igf-1 was added, only 253 genes were upregulated and 132 downregulated compared to CTR myoblasts (Figure 4 and Figure 5). We also found 182 and 92 genes commonly upregulated and downregulated in response to AA and Igf-1, respectively (Figure 5).

**Figure 6:**
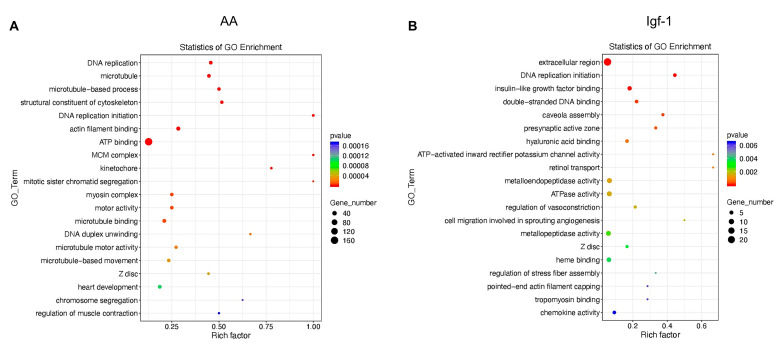
Global Gene Ontology (GO) enrichment analysis of the genes that significantly changed their transcription in response to AA (**A**) or Igf-1 (**B**). The size of the dots represents the number of genes present in each GO term, while the color indicates the *p*-value associated with each GO term identified. The name of the enriched GO term is indicated on the left side of the panel, whereas the GO Rich Factor (ratio of the number of differentially expressed genes in the pathway to the total number of genes in the pathway) is indicated in the lower part of each panel.

The Gene Ontology analysis of the up and downregulated genes in response to the different treatments showed differences between the processes affected and their intensity. Several GO terms related to DNA replication and cell cycle (0007049; 0006260; 0003688), muscle differentiation (0042692; 0003012), and sarcomere and muscle cytoskeleton (0007010; 0045214; 0008092; 0043292; 0030017) were upregulated in response to AA; while GO terms such as transport activity (0034219; 0015293) or growth factor and cytokine activity (0008083; 0005125) were downregulated in this condition (Table 1). The addition of Igf-1 increased the expression of genes related to muscle development (0042692; 0055001; 0061061) and muscle cytoskeleton (0030016; 0030017; 0015629) (Table 1). Some GO terms were shared between AA and Igf-1, but the number of genes involved was significantly different, with many more genes modified by AA (Table 1; Figure 6).

### 2.3. Transcriptomic Analysis of ncRNAs

**Figure 7:**
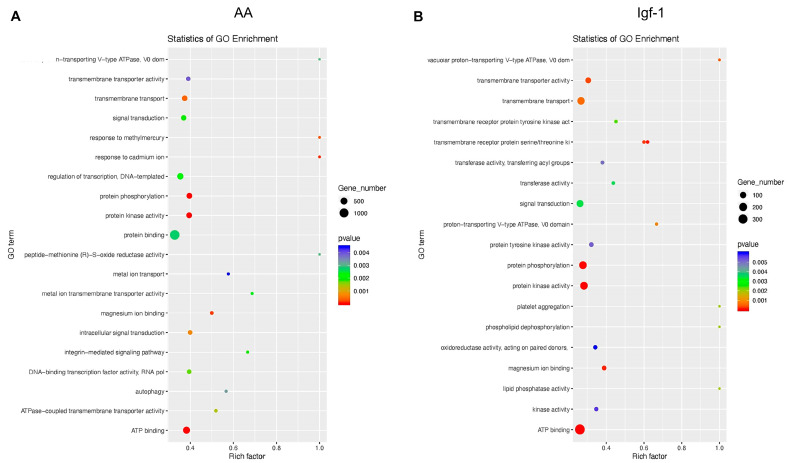
Global Gene Ontology (GO) enrichment analysis of the miRNAs that significantly changed their transcription in response to AA (**A**) or Igf-1 (**B**). The size of the dots represents the number of genes present in each GO term, while the color indicates the *p*-value associated with each GO term identified. The name of the enriched GO term is indicated on the left side of the panel, whereas the GO Rich Factor (ratio of the number of differentially expressed genes in the pathway to the total number of genes in the pathway) is indicated in the lower part of each panel.

The total number of ncRNAs affected by the treatments was significantly smaller compared to the mRNAs. A total of 54 miRNAs were significantly upregulated in response to AA, such as *miR-1* (log2FC = 2.62), *miR-133a/b* (log2FC = 2.54), *miR-181b* (log2FC = 1.80), *miR-499* (log2FC = 1.54) or *miR-206* (log2FC = 1.48); and 26 miRNAs were downregulated in response to AA, including *miR-29d* (log2FC = −2.79), *miR-203a/b* (log2FC = −1.38) or *miR-122* (log2FC = −0.77) (Figure 4 and Figure 5; Supplementary File S3). Gene Ontology analysis based on human miRNA–mRNA interactions showed that miRNA modified by the presence of AA might control mRNA involved in protein and ATP binding and regulation of transcription (Figure 7). On the other hand, in response to Igf-1, only 20 miRNAs significantly increased their expression in response to Igf-1, such as *miR-27c* (log2FC = 1.67), *miR-1* (log2FC = 1.56), *miR-19a/b* (log2FC = 1.06), or *miR-133a/b* (log2FC = 0.77); and a total of 26 miRNA appeared downregulated but most of them with a change log2FC < −1, such as *miR-203a/b* (log2FC = −0.83), *miR-128* (log2FC = −0.83); *miR-122* (log2FC = −0.71), *miR-206* (log2FC = −0.72), *miR-27a* (log2FC = −0.48) and *miR-221* (log2FC = −0.23) (Figure 4 and Figure 5; Supplementary File S3). Gene Ontology analysis based on human data predicted that those miRNAs controlled mRNAs involved in transmembrane transport, protein phosphorylation, signal transduction, and ATP binding (Figure 7).

The number of lncRNAs significantly modified was also small compared to mRNAs and miRNAs. In response to AA, only 17 lncRNAs appeared to be significantly upregulated with a log2FC between 1 and 2 (Figure 4 and Figure 5; Supplementary File S1). We also found 13 lncRNAs significantly downregulated in response to the presence of AA, showing a log2FC between −1 and −5. In response to Igf-1, only 4 lncRNAs were significantly increased with log2FC between 1.05 and 1.70. Similarly, only 7 lncRNAs appeared to be significantly downregulated in response to Igf-1 with log2FC between −1.20 and −12.40 (Figure 4 and Figure 5; Supplementary File S1). Due to the lack of information about GO terms associated with fish lncRNAs, no GO enrichment analysis was performed.

### 2.4. Predicted Interactions of miRNAs and lncRNAs with mRNAs Based on Transcriptomic Correlations and Bioinformatics Analysis

To better understand the changes in response to AA and Igf-1, correlation and binding analyses were performed between miRNAs, lncRNAs, and mRNAs. Significantly modified miRNAs, lncRNAs, and mRNAs were considered candidates for further consideration when correlations had a negative Pearson index lower than −0.80. We found up to 14,658 negative correlations between miRNAs and mRNAs and a total of 7488 negative correlations between significantly modified lncRNAs and mRNAs using all treatments (Supplementary File S4), indicating the possibility of co-regulation. To further investigate how miRNAs and lncRNAs might be involved in the variations of transcription observed in mRNAs, we estimated the probability of direct interaction between miRNAs or lncRNAs and mRNAs with a correlation lower than −0.80 using bioinformatic tools. While several strong interactions (<−25 kcal/mol) were found in response to AA (Supplementary File S5), only a handful of miRNAs dominate the majority of interactions observed, such as *miR-17a*, *miR-128*, *miR-133a/b* and *miR-206*. Similarly, in response to Igf-1, we found some miRNAs predicted to interact with multiple mRNAs, such as *miR-34*, *miR-221*, and *miR-338* (Supplementary File S5). Gene Ontology enrichment analysis of the mRNAs predicted to both possibly correlate and interact with miRNAs was performed to determine the biological processes regulated by them. In the case of the AA treatment, miRNAs were involved in the downregulation of genes related to Igf binding, development, protein catabolism, sarcomere production, and DNA replication (Table 2). In the Igf-1 treatment, miRNAs were involved in the possible regulation of mRNAs related to the extracellular region and upregulation of genes related to development, DNA metabolic process, and cytoskeleton (Table 2).

We also found strong negative correlations between lncRNAs and those mRNAs significantly modified by treatments (Supplementary File S4), but only 8 lncRNAs simultaneously showed strong negative correlations (ρ < −0.80) and significant interactions (ndG < −0.10 kcal/mol) with some of the mRNAs identified to change in response to treatments such as *acta1*, *rbm24b*, *h2az1*, *pin1*, *tcima*, *psmb3*, *tnni2*, *nupr1a*, *rgcc*, and *igfbp6a* (Supplementary Table S1, Supplementary File S6).

The possibility of lncRNAs regulating mRNAs abundance by acting as miRNAs sponges was also investigated. Correlations of <−0.80 between lncRNAs and miRNAs were considered possible candidates (Table 3). From those, we found 30 lncRNAs with strong predicted interactions with miRNAs, which in turn possibly regulate multiple mRNAs, such as *ENSSAUG00010001802* (interacting with *miR-27a*, *miR-29d* and *miR-29b*), *ENSSAUG00010012228* (interacting with *miR-338*, *miR-133a*/b, *miR-17a*, *miR-125a*, *miR-106*, *miR-217*, and *miR-206*) or *ENSSAUG00010017089* (interacting with *miR-206*, *miR-106*, *miR-128*, and *miR-17a*) (Table 3).

## 3. Discussion

Understanding the regulation of fish muscle development and growth is necessary to optimize aquaculture production because it is the most valuable part of the fish for the aquaculture industry. To thoroughly study the mechanisms orchestrating the myogenesis process, it is necessary to consider the complex networks integrating not only the transcription of genes but also of ncRNAs like miRNAs and lncRNAs [23,73]. For this purpose, fish myoblast cell culture is a very useful and powerful tool that allows the analysis of many signaling pathways and molecular networks under controlled conditions [74]. In this study, a cell culture of gilthead sea bream myoblasts was used to explore for the first time in fish the transcriptional response of mRNAs, miRNAs, and lncRNAs in response to AA and Igf-1, as well as their possible regulatory network.

Both pro-growth signals induced many transcriptional changes compared to untreated cells, but the AA group showed a higher number of transcriptionally modified mRNAs compared to Igf-1 (Figure 4). These results are in agreement with previous studies in pacu (*Piaractus mesopotamicus*) [25] and Atlantic salmon (*Salmo salar*) [75] that showed a better capacity of AA compared to Igf-1 alone to boost myoblast response, suggesting that the Igf-1 might need the assistance of AA to perform its function. Studies in L6 murine muscle cell lines have shown that blocking Igf-1 expression did not decrease the protein synthesis rate when induced by AA, indicating that Igf-1 transcription is a covariate to AA initiation of protein synthesis through an unknown process [76]. It is well known that Igf-1 performs its functions through the phosphorylation of Akt, which leads to the promotion of cell proliferation and protein synthesis by activating the mTOR complex 1 (mTORC1) [17,77,78]. The activation of mTORC1 can also be triggered by AA, but in this case is done through the Ragulator complex, a system believed to act independently of the Akt pathway [11,79,80]. Although it is presumed that the activation of mTORC1 by AA and Igf-1 occurs in an independent way, it might be possible that the lack of AA impairs the activation of this complex by the Igf-1/Akt pathway through a not yet described mechanism that needs further investigation.

Furthermore, there was a clear difference in the magnitude of transcriptional changes induced by both treatments: the upregulation of genes such as *myoz1b*, *stac3*, *tnnt2c*, *igfbp2a*, or *usp28* was much higher in response to AA than in response to Igf-1, while downregulated genes such as *plvapb*, *ccn5*, or *cav2* had their transcription less reduced in response to Igf-1 compared to AA. It is important to highlight that all these genes participate in the regulation of muscle growth by modulating mechanisms related to myogenesis and protein balance in the muscle fiber [8,81,82,83]. For instance, the upregulation of *myoz1b*, *stac3*, and *tnnt2c* at day 9 of culture with AA and Igf-1 confirms the correct development of myogenesis under these treatments because they are genes that encode for proteins involved in muscle contraction and are expected to increase their expression when myoblasts are fusing to form myotubes [82].

It is interesting to highlight that despite the big differences in the number of mRNAs modified and the magnitude of the changes, when GO analysis was performed for up and downregulated genes, both treatments regulated common processes related to muscle growth, differentiation, and sarcomere formation. This fact suggests that both AA and Igf-1 were able to promote the transcription of components of the molecular network controlling protein synthesis and sarcomere development. Moreover, it seemed that both treatments were able to increase DNA replication and cell proliferation (Figure 6).

Regarding the ncRNAs, we identified a comprehensive repertoire of miRNAs and lncRNAs present in gilthead seabream myoblasts with potential roles in regulating muscle growth. We found that the most expressed miRNAs in the gilthead sea bream myoblasts were *miR-21*, *miR-146*, *miR-22b*, and *miR-206*, with only the last one being a canonical myomiR [43,84], although the rest are also known to have roles on the control of skeletal muscle growth. For instance, in mammalian models, *miR-21* is known to downregulate the transcription of *pten* [85,86], a component of the mTOR network, but also *col1a1*, *col6a*, and *tgf-ß*, components of the extracellular matrix [87]. On the other hand, *miR-146* is known to promote myoblast differentiation through the regulation of *smad4*, *notch1*, and *hmga2* [88], and *miR-22b* is also involved in myoblast differentiation by targeting *tgfßr1* [89]. It is not surprising that these miRNAs promoting differentiation were highly expressed, considering that we used myoblasts developed for 8 days when myoblasts are slowing down proliferation and entering into the differentiation program, where TGF-ß is known to inhibit differentiation [90,91]. We only found a significant decrease in *tgfb3* expression (FDR = 0) in response to AA (log2FC = −1.55), and less modulated in response to Igf-1 (log2FC = −0.90). Other components of the TGF-ß pathway, such as *tgfb2*, *tgfb5*, *tgfb3*, and *tgfb1a*, were non-significantly downregulated in response to both treatments.

Like mRNAs, more miRNAs changed their transcription in response to AA compared to Igf-1 (Figure 4). Not many miRNAs were downregulated by the pro-growth treatments, but we found low expression of *miR-22b* (when upregulated promotes differentiation) [89], *miR-206* (promotes differentiation) [92], *miR-221* (involved in proliferation and differentiation) [93] and *miR-338* (function not known, but is differentially expressed in skeletal muscle of different species under different growth conditions) [25,94,95] in response to Igf-1. The fact that some differentiation-inducing miRNAs identified in mammals [21,22,43] appeared to be downregulated in the present experiment seems to be at odds with the results obtained, which suggests that both proliferation and differentiation were stimulated (as indicated by the GO enrichment analysis). However, we also found a significant increase of miRNAs that promote differentiation such as *miR-1* (log2FC > 1.5; increased in response to both treatments), *miR-206* (log2FC = 1.48; increased with AA), *miR-499* (log2FC = 1.54; promotes differentiation toward slow phenotype, increased with AA), *miR-181* (log2FC = 1.8 increased with AA) and *miR-34* (log2FC = 1, inhibits proliferation, increased with Igf-1). At the same time, an upregulation of miRNAs generally associated with myoblast proliferation was also observed in response to AA, such as *miR-128* (log2FC ≥ 0.78), or in response to both treatments, like the *miR-133a/b* (log2FC ≥ 0.66). The transcriptional changes of miRNAs and mRNAs involved in both myogenic proliferation and differentiation are likely due to the fact that the cell cultures used in the present study contain a mixture of cells at different stages, with still proliferative myoblasts but most cells differentiating.

Our analysis showed strong correlations between miRNAs and mRNAs differentially expressed in response to the treatments. However, many of the identified correlations (<−0.80) had relatively low predicted interactions (<−25 kcal/mol), suggesting that the mRNAs and miRNAs might be part of the same networks but not directly regulating each other. The strong correlations and significant interactions found were dominated by a small number of miRNAs: *miR-133*, *miR-128* or *miR-206* (upregulated) and *miR-27a*, *miR-92a* or *miR-29d* (downregulated) in the AA treatment; *miR-128*, *miR-125*, *miR-338*, *miR-206* or *miR-27a* (downregulated) and *miR-34* or miR-7147 (upregulated) in the Igf-1 treatment. The percentage of genes whose transcription seems to be potentially regulated by miRNAs was relatively low. However, we must take into consideration that in the present study, we have used quite stringent conditions, reducing the number of interactions identified. Likewise, the correlations were performed with only nine samples, and the strength of such correlations must be considered cautiously.

Unraveling the roles of lncRNAs in fish skeletal muscle based on transcriptomic data is quite challenging, and we can only hypothesize their possible functions using bioinformatic approaches. The study of lncRNAs in mammals has revealed their importance in the transcriptomic regulation of muscle development, and some lncRNAs have been shown to be critical in the control of muscle gene expression, including the *linc-RAM* (enhances myogenic differentiation by interacting with MyoD) [63], *MUNC* (increases *MyoD*, *Myogenin*, and *Myh3* mRNAs and facilitates the function of MyoD) [61,62], *OIP5-AS1* (interacts with *MEF2C* mRNA and promotes myogenic gene expression) [96], or *Lnc-31* (binds to *Rock1* mRNA and sustains myoblast proliferation) [97]. Similarly, lncRNAs can also exert their functions directly interacting with miRNAs, such as *linc-MD1* and *MDNCR* (interact with *miR-133*) [98,99], *Sirt1* AS (interacts with *miR-34a*) [100] or *linc-smad7* (interacts with *miR-125b*) [101], acting as miRNAs sponges [102]. However, it is very difficult to translate the research done in mammalian models to other species due to the low degree of conservation found between lncRNAs [72]. Our data indicates that only a small fraction of the lncRNAs identified responded to the pro-growth signals, with most of them showing low expression, as previously observed in other studies [55,103]. It is interesting to notice that many of the lncRNAs previously identified in gilthead sea bream skeletal muscle [24] had very low levels of expression in myoblasts developed for 8 days, although one of them, the *ENSSAUG00010020194*, slightly increased transcription in response to pro-growth signals (log2FC < 1), but not significantly. Similarly, its predicted target (*myod1*) also slightly changed its transcription (log2FC < 1) in response to pro-growth signals, but not significantly.

Furthermore, our analysis revealed that a higher number of lncRNAs simultaneously exhibit strong negative correlations and interactions with miRNAs (Table 3) compared to mRNAs (Supplementary Table S1), which changed in response to the treatments. This fact may suggest that the contribution of lncRNAs to the modulation of transcription might be done mainly as miRNAs sponges rather than through direct interactions with mRNAs. Among the miRNAs that negatively correlate and interact with lncRNAs are those associated with multiple mRNAs modified by treatments: *miR-338*, *miR-92*, *miR-34 miR-206*, *miR-133*, *miR-7147*, *miR-27*, *miR-29*, *miR-125* and *miR-128* (Table 3). This indicates that highly expressed lncRNAs bind to the miRNAs, preventing the degradation of target mRNAs, which appear increased (and vice versa). Supplementary Figures S1 to S3 show examples of possible networks of mRNAs, miRNAs, and lncRNAs controlling some biological processes in response to AA and Igf-1. For example, Supplementary Figure S2A exposes a group of genes involved in muscle development that were upregulated with AA and could be affected by some miRNAs (*miR-27a*, *miR-29d*, *miR-92a*) that, in turn, might be sequestered by specific lncRNAs acting as sponges. These figures show part of the distinct levels in the transcriptional regulation and illustrate the complexity behind the interactions between different molecules.

Moreover, it is interesting to note that some of the interactions found in our study are also predicted for some human lncRNAs, such as *linc-MD1* (*miR-133*) [98], *Sirt1 AS* (*miR-34*) [100], or *lnc-mg* (*miR-125*) [104]. The results suggest that some roles as sponges of lncRNAs in muscle might be conserved in teleost fish. However, it is important to highlight that we have found a relatively low conservation between lncRNAs with similar interactions in fish and mammals. For instance, *ENSSAUG00010016143* (which interacts with *acta1*; Supplementary Table S1) had a 44% similarity with *Myolinc* [105] and not quite a good alignment, and the majority of lncRNAs identified to interact with *miR-133* have less than 30% similarity with *linc-MD1*. Similarly, we did not find any clear conservation of the synteny between mammalian and fish lncRNAs with conserved targets, suggesting that while lncRNA interactions might be conserved, their evolution history is not clear.

Overall, this work is the first step in the identification of the network of mRNAs, miRNAs, and lncRNAs controlling muscle development and growth in gilthead sea bream, pointing out potential candidates with a high confidence value that might be of great interest for further experimental work. Moreover, this study contributes to a better understanding of the modulation of mRNAs and ncRNAs transcription by AA and Igf-1, along with their potential regulatory mechanisms in this species, and establishes the basis for future research focusing on the possible dose-dependent response of these pro-growth signals and exploring their synergistic effects.

## 4. Materials and Methods

### 4.1. Gilthead Sea Bream Primary Myoblast Cell Culture and Treatments

Myoblasts were isolated and cultured according to the protocol described by Fauconneau and Paboeuf (2000) [106] and adapted to gilthead sea bream by Montserrat et al. (2007) [107]. Briefly, fast-twitch muscles were collected from the epaxial region of gilthead sea bream fingerlings (≈ 5 g) and mechanically dissociated with scalpels, enzymatically digested with 0.2% collagenase type IA (Ref. C9891) and 0.1% trypsin (Ref. T4799), filtered with cell strainers (Ref. CLS431752 and CLS431750), centrifuged, resuspended and plated in poly-L-lysine/laminin (Ref. P6282 and L2020) pre-treated 6-well plates (Ref. 140675) with complete growth medium [DMEM (Ref. D7777), 9 mM NaHCO_3_ (Ref. S5761), 20 mM HEPES (Ref. H3375), 1.1 g/L NaCl (Ref. S5886), 1% antibiotic/antimycotic (Ref. A5955), and 10% fetal bovine serum (FBS; Ref. F7524), pH 7.4], at a density of 1.5 × 10^6^ cells/well. All media, reagents, and cell strainers were obtained from Sigma-Aldrich (Tres Cantos, Madrid, Spain), and the culture plates were obtained from Thermo Fisher Scientific (Sant Cugat del Vallès, Barcelona, Spain). Myoblasts were incubated at 22 °C, with a full replacement of the culture medium every day. Myoblasts morphology was monitored regularly under an inverted microscope (Carl Zeiss, Oberkochen, Germany) and let to develop until the first myoblast fusion events were visible (around day 8 of culture). The present work was based on 3 independent cell cultures.

On day 8 of culture, myoblasts were incubated for 12 h in a free AA medium [Earle’s balanced salt solution 1× (Ref. E7510), 9 mM NaHCO_3_, 20 mM HEPES, 1.1 g/L NaCl, Vitamin Mix 1× (Ref. M6895), 1% antibiotic/antimycotic, and 4 g/L D-glucose (Ref. G8270)] to reduce gene expression to basal levels. Cells were incubated for additional 24 h in free AA medium (CTR group), medium with AA (AA group; DMEM, 9 mM NaHCO_3_, 20 mM HEPES, 1.1 g/L NaCl, and 1% antibiotic/antimycotic), or medium with recombinant Igf-1 (Igf-1 group) [free AA medium supplemented with Igf-1 from gilthead sea bream at 100 ng/mL (Ref. CYT-295, ProSpec, Rehovot, Israel), and 0.1 mg/mL of RIA grade bovine serum albumin (Ref. A7030, Sigma-Aldrich) as carrier protein]. The treatments were performed according to the protocol described by Bower and Johnston (2010) [75] and Garcia de la serrana and Johnston (2013) [108].

### 4.2. RNA Extraction, Sequencing, and Bioinformatic Analyses

After the treatments, gilthead sea bream myoblasts were washed thrice with PBS following medium removal. Total RNA was extracted using Trizol (Ref. 15596026, Thermo Fisher Scientific), followed by chloroform, isopropanol, and ethanol extraction as recommended by the manufacturer. Total RNA was resuspended in RNase-free water, and its concentration and integrity were estimated by spectrophotometry using Nanodrop 2200^TM^ (Thermo Fisher Scientific) and a 1% (*w*/*v*) agarose gel, respectively.

The generation of DNA libraries and sequencing of mRNAs and miRNAs were performed by LC Sciences (Houston, TX, USA). Transcriptome was obtained through the NovaSeq 6000 platform (Illumina, San Diego, CA, USA) with 150 base pairs, paired-end, and 6 GB data per sample (40–50 million reads). microRNAome was obtained through the HiSeq 4000 platform (Illumina, San Diego, CA, USA) with 50 base pairs, single-end, and 10 million reads per sample. For transcriptome analysis, adapters and low-quality reads were removed using an in-house perl script and then mapped against the latest gilthead sea bream genome available (www.ensembl.org/index.html; accessed on 15 January 2023) using HISAT2 software v.2.2.1 [109]. Transcripts were assembled, followed by mRNA expression profiling analysis using StringTie v.2.2.0 [110] and expressing the results as FPKM (fragments per kilobase of exon per million fragments mapped). For the microRNAome, adapters and low-quality reads were removed using in-house perl scripts. Subsequently, unique sequences with length in 18–26 nucleotides were mapped to specific species precursors in miRBase 22.0 (www.mirbase.org, accessed on 18 December 2022) by BLAST search to identify known miRNAs and novel 5p- and 3p- derived miRNAs candidates. The remaining sequences were mapped to other selected species precursors (with the exclusion of specific species) in miRBase v.22.1 by BLAST search, and the mapped pre-miRNAs were further BLASTed against the specific species genomes to determine their genomic locations.

Gene Ontology (GO) analysis was performed using the STRING online tool against the zebrafish (*Danio rerio*) database (https://string-db.org/, accessed on 18 December 2022). Venn diagrams were obtained using plotting software (https://pnnl-comp-mass-spec.github.io/Venn-Diagram-Plotter/, v.1.6.7458, accessed on 20 July 2023).

Pearson correlation analysis was carried out using RStudio v.1.1.419 [111] to detect correlations between mRNAs-miRNAs and lncRNAs-miRNAs differentially expressed in response to the treatments. Sequences’ interactions were predicted using RNAhybrid v.2.2.1 (https://bibiserv.cebitec.uni-bielefeld.de/rnahybrid, accessed on 15 January 2023) [112], with a minimum free energy (MFE) threshold of <−25 kcal/mol. Possible interactions between lncRNAs and mRNAs were explored using LncTar software (www.cuilab.cn/lnctar, accessed on 15 January 2023), with a threshold of normalized binding free energy (ndG) < −0.10.

### 4.3. Validation of RNA-Seq Results by qPCR

To validate the expression profiles from the RNA-Seq analysis using qPCR, we selected mRNAs, miRNAs, and lncRNAs that showed significant differences between the experimental groups in the RNA-Seq analysis. We used samples of the three experimental conditions (CTR, AA, and Igf-1, explained in Section 4.1) from six independent cell cultures. Total RNA was extracted as previously described (Section 4.2). The qPCR analyses were carried out following the MIQE guidelines [113] in a CFX384™ Real-Time System (Bio-Rad, El Prat de Llobregat, Barcelona, Spain). The analysis was performed in triplicate, using for each well: 2.5 µL of iTAQ Universal SYBR^®^ Green Supermix (Ref: 1725125, Bio-Rad), 1 µL of cDNA, 250 nM (final concentration) of forward and reverse primers and 1.25 µL of DEPC water. The reaction protocol was: 3 min at 95 °C, 40 × (10 s at 95 °C, 30 s at the annealing temperature of the primers, and fluorescence detection), followed by an amplicon dissociation analysis. In the case of the miRNAs, we designed primers to amplify pri-miRNAs sequences, to distinguish between the expression of different paralogs that have similar mature sequences. The genes analyzed were *igfbp6*, *cav3*, *trim63*, *acta1*, *stac3*, *usp28*, *myoz1b*, *cpt1b*, *wnt4*, and two reference genes, *rps18* and *tomm20b*. The pri-miRNAs were *pri-miR-1-2*, *pri-miR-133a-1*, *pri-miR-133a-2*, *pri-miR-133b*, *pri-miR-29a*, *pri-miR-206*, *pri-miR-221*, and *pri-miR-222*. The lncRNAs were *ENSSAUG00010012549*, *ENSSAUG00010001802*, *ENSSAUG00010004711*, and *ENSSAUG00010020194*. The transcript abundance was calculated using the Bio-Rad CFX Manager™ 3.1 software, relative to the geometric mean of the reference genes [114]. Statistical analyses were performed using IBM SPSS Statistics v. 25 (IBM Corp., Armonk, NY, USA). The normality and homoscedasticity of the data were checked with a Shapiro–Wilk test and a Levene’s test, respectively. Groups were compared using one-way ANOVA followed by a Tukey’s post hoc test (significant differences considered at *p*-value < 0.05). All raw and processed data from these analyses and the primers used for the qPCRs are shown in Supplementary File S7. Transcript levels of genes, pri-miRNAs, and lncRNAs showed concordance between RNA-Seq and qPCR results, revealing similar expression patterns in both cases.

### 4.4. Statistics of RNA-Seq Data

Differences in transcription levels between treatments obtained from RNA-Seq data were biologically relevant when log2-fold change (log2FC) was ≤−1 and ≥1 and the corrected *p*-value (False Discovery Rate, FDR) was ≤0.05. In the case of miRNA-Seq data, only the FDR threshold was considered. For Gene Ontology analysis, differences between categories were compared against the zebrafish database and considered significant when FDR < 0.05. All graphs were generated using ggplot2 [115].

## 5. Conclusions

In summary, both AA and Igf-1 treatments induced the transcription of components related to myogenesis (proliferation and differentiation), sarcomere formation, and protein synthesis, but AA appeared to have a greater impact on the transcriptional response of genes and ncRNAs compared to Igf-1. Some of the miRNAs most regulated by the pro-growth signals were canonical myomiRs with known roles in myogenic mechanisms, such as *miR-1*, *miR-133a/b*, and *miR-206*, but also other miRNAs with more unknown functions in muscle, such as *miR-203a/b* or *miR-122*. In contrast, few lncRNAs responded to the treatments, with most of them showing very low expression, but interestingly, our study suggests that the lncRNAs act mainly as miRNAs sponges in response to AA and Igf-1. Furthermore, the results of the correlations and predicted interactions between mRNAs, miRNAs, and lncRNAs point out the importance and complexity of the network controlling muscle development and growth in response to pro-growth signals in gilthead sea bream fast muscle myoblasts. These results will serve as significant resources for future studies that further investigate the role of ncRNAs in the myogenesis processes of teleost.

## Figures and Tables

**Table 1:** Gene Ontology analysis of the up and downregulated genes in response to AA and Igf-1.

AA vs. CTR				
		GO Term	Description	FDR
Upregulated	*Biological Process*	0007049	Cell cycle	5.87 × 10^−45^
		0006260	DNA replication	2.12 × 10^−19^
		0007010	Cytoskeleton organization	4.82 × 10^−08^
		0003012	Muscle system process	1.87 × 10^−07^
		0042692	Muscle cell differentiation	3.52 × 10^−05^
		0045214	Sarcomere organization	4.19 × 10^−07^
	*Molecular Function*	0008092	Cytoskeletal protein binding	1.61 × 10^−10^
		0003688	DNA replication origin binding	3.47 × 10^−07^
		0005515	Protein binding	8.75 × 10^−06^
		0005524	ATP binding	0.0074
		0016787	Hydrolase activity	0.026
	*Cellular Component*	0043232	Intracellular non-membrane-bounded organelle	2.53 × 10^−30^
		0043292	Contractile fiber	2.15 × 10^−18^
		0030017	Sarcomere	6.87 × 10^−17^
		0005654	Nucleoplasm	0.0279
Downregulated	*Biological Process*	0032870	Cellular response to hormone	0.0043
		0043473	Pigmentation	0.008
		0034219	Carbohydrate transmembrane transport	0.0215
	*Molecular Function*	0015293	Symporter activity	0.0088
		0008083	Growth factor activity	0.0430
		0005125	Cytokine activity	0.0430
		0005539	Glycosaminoglycan binding	0.0430
	*Cellular Component*	0005576	Extracellular region	0.0110
		0110165	Cellular anatomical entity	0.0110
		0031082	BLOC complex	0.0416
**Igf-1 vs. CTR**				
Upregulated	*Biological Process*	0042692	Muscle cell differentiation	0.0026
		0055001	Muscle cell development	0.0120
		0061061	Muscle structure development	0.0120
		0009987	Cellular process	0.0120
	*Cellular component*	0030016	Myofibril	0.00029
		0030017	Sarcomere	0.00029
		0099512	Supramolecular fiber	0.00029
		0015629	Actin cytoskeleton	0.0074
Downregulated	*Molecular Function*	0005539	Glycosaminoglycan binding	0.0250
	*Cellular Component*	0005576	Extracellular region	0.0003

FDR: False discovery rate.

**Table 2:** Gene Ontology enrichment analysis of the up and downregulated genes that were predicted to correlate and interact with miRNAs.

		GO Term	Description	FDR
AA vs. CTR				
Upregulated	*Biological Process*	0009888	Tissue development	0.034
		0030163	Protein catabolic process	0.034
		0097435	Actin cytoskeleton organization	0.034
		0006260	DNA replication	0.034
	*Molecular Function*	0004298	Threonine-type endopeptidase activity	0.001
	*Cellular Component*	0005622	Intracellular	0.0007
		0032991	Protein-containing complex	0.0086
Downregulated	*Biological Process*	0043473	Pigmentation	0.0002
		0019262	N-acetylneuraminate catabolic process	0.026
	*Molecular Function*	0016798	Hydrolase activity, acting on glycosyl bonds	0.031
		0005520	Insulin-like growth factor binding	0.041
	*Cellular Component*	0110165	Cellular anatomical entity	0.010
		0012505	Endomembrane system	0.039
		0005773	Vacuole	0.000
**Igf-1 vs. CTR**				
Upregulated	*Biological Process*	0048731	System development	0.045
		0006259	DNA metabolic process	0.045
		0055001	Muscle cell development	0.008
	*Cellular Component*	0005856	Cytoskeleton	0.019
		0030017	Sarcomere	0.000
Downregulated	*Cellular Component*	0005576	Extracellular regions	0.000

FDR: False discovery rate.

**Table 3:** Potential lncRNAs acting as miRNAs sponges. Predicted interactions between lncRNAs and miRNAs significantly modified in response to AA and Igf-1.

lncRNAs ID	miRNAs	Correlation Index	Energy (ndG)
*ENSSAUG00010001802*	*miR-27a*; *miR-29d*; *miR-29b*	−0.88; −0.86; −0.87	−29.1; −26.2; −27.1
*ENSSAUG00010017848*	*miR-122*; *miR-92a*; *miR-29a*; *miR-29d*; *miR-29b*; *miR-203a*; *miR-25*; *miR-31*	−0.88; −0.80; −0.86; −0.92; −0.84; −0.91; −0.84; −0.81	−26.7; −32.5; −32.2; −32.2; −26.6; −28.1; −27.7; −28.6
*ENSSAUG00010024948*	*miR-122*; *miR-92a*; *miR-10c*; *miR-10d*; *miR-27a*; *miR-29b*; *miR-31*	−0.92; −0.89; −0.87; −0.87; −0.82; −0.90; −0.93	−26.0; −27.6; −25.5; −25.5; −30.6; −32.3; −26.0
*ENSSAUG00010012228*	*miR-338*; *miR-133a*; *miR-133b*; *miR-206*; *miR-17a*; *miR-125a*; *miR-106*; *mir-217*	−0.80; −0.92; −0.91; −0.87; −0.80; −0.82; −0.90; −0.91	−27.1; −26.1; −26.1; −28.6; −27.8; −28.6; −28.1; −29.0
*ENSSAUG00010000237*	*miR-125b*	−0.83	−28.5
*ENSSAUG00010012182*	*miR-7a*; *miR-338*; *miR-133a*; *miR-133b*; *miR-206*; *miR-106*; *miR-17a*; *miR-125a*	−0.93; −0.80; −0.92; −0.91; −0.87; −0.90; −0.80; −0.82	−29.0; −27.1; −26.1; −26.1; −28.6; −28.6; −27.8; −28.6
*ENSSAUG00010012549*	*miR-17a*	−0.80	−26.5
*ENSSAUG00010015941*	*miR-206*; *miR-17a*; *miR-125b*; *mir-145*; *miR-454*	−0.86; −0.83; −0.84; −0.83; −0.86	−26.4; −30.2; −30.2; −25.6; −29.7
*ENSSAUG00010016074*	*miR-15a*; *miR-19b*; *miR-217*; *miR-34*	−0.89; −0.81; −0.85; −0.81	−27; −25.3; −27.6; −27.5
*ENSSAUG00010016143*	*miR-133a*	−0.82	−25.1
*ENSSAUG00010017089*	*miR-206*; *miR-106*; *miR-128*; *miR-17a*	−0.95; −0.82; −0.88; −0.91	−28.6; −26.8; −30.7; −27.8
*ENSSAUG00010016280*	*miR-122*; *miR-92a*; *miR-25*	−0.86; −0.81; −0.80	−29.6; −27.8; −25.1
*ENSSAUG00010002983*	*miR-15a*	−0.90	−31.2
*ENSSAUG00010008657*	*miR-338*; *miR-15a*; *miR-34*; *miR-7147*	−0.88; −0.85; −0.88; −0.90	−33.3; −29.4; −25.6; −25.8
*ENSSAUG00010022074*	*miR-128*; *miR-365*; *miR-454*; *miR-19a*; *miR-15a*; *miR-34*; *miR-7147*	−0.83; −0.83; −0.82; −0.82; −0.80; −0.83; −0.86	−31.3; −29.7; −25.9; −27.5; −32.9; −28.6; −27.2
*ENSSAUG00010013187*	*miR-30e*; *miR-29a*; *miR-29d*; *miR-22b*; *miR-30a*	−0.91; −0.90; −0.83; −0.80; −0.90	−26.8; −28.8; −28.8; −28.3; −29.5
*ENSSAUG00010013622*	*miR-30e*; *miR-29d*; *miR-8160ba*; *miR-30a*	−0.85; −0.84; −0.90; −0.82	−27.0; −27.3; −25.2; −26.5;
*ENSSAUG00010015504*	*miR-27d*; *miR-30a*	−0.85; −0.81	−29.1; −30.1
*ENSSAUG00010016109*	*miR-30e*; *miR-25*; *miR-27d*; *miR-27a*	−0.81; −0.82; −0.84; −0.85	−31.7; −25.8; −25.5; −26.0
*ENSSAUG00010001416*	*miR-29b*	−0.83	−25.2
*ENSSAUG00010017066*	*let-7g*	−0.81	−28.0
*ENSSAUG00010002786*	*miR-10926*; *miR-29d*; *miR-8160ba*	−0.80; −0.83; −0.97	−28.1; −28.7; −26.3
*ENSSAUG00010004711*	*miR-8160ba*	−0.89	−27.1
*ENSSAUG00010026349*	*miR-10926*; *miR-22b*; *miR-29a*; *miR-29d*; *miR-551*; *miR-8160ba*	−0.81; −0.82; −0.82; −0.82; −0.81; −0.82	−29.4; −27; −26.2; −26.2; −25.6; −25.6
*ENSSAUG00010009596*	*miR-128*; *mir-365*; *miR-7550*	−0.95; −0.90; −0.83	−27.7; −27.3; −25.5
*ENSSAUG00010015789*	*miR-128*; *miR-365*; *miR-125b*	−0.89; −0.82; −0.81	−28.9; −32.7; −25.2
*ENSSAUG00010020704*	*miR-128*; *miR-365*; *miR-26b*; *miR-454*; *miR-19a*; *miR-15a*; *miR-34*; *miR-7147*	−0.83; −0.83; −0.86; −0.82; −0.82; −0.80; −0.83; −0.85	−31.3; −29.2; −26.3; −25.9; −27.5; −32.9; −28.6; −27.2
*ENSSAUG00010010920*	*miR-139*; *miR-27d*; *miR-8160ba*	−0.81; −0.85; −0.83	−28.5; −26.1; −25.9
*ENSSAUG00010003663*	*miR-15a*; *miR-301b*; *miR-33b*; *miR-34*; *miR-7147*	−0.91; −0.81; −0.81; −0.84; −0.85	−29.1; −25.3; −26.1; −33.2; −28.1
*ENSSAUG00010016209*	*miR-27a*; *miR-122*; *miR-92a*	−0.85; −0.86; −0.81	−26.0; −29.6; −27.8

The predicted interactions between lncRNAs and miRNAs shown are based on transcriptional correlations and bioinformatics analysis. Interactions with Pearson correlations lower than −0.80 and with predicted interaction energies lower than −25.0 kcal/mol are shown.

## Data Availability

The data presented in this study are available in the current article and its corresponding Supplementary material. The raw and processed data of transcriptome and microRNAome analyses have been deposited on the Gene Expression Omnibus (GEO) DataSets, under the accession number GSE246665.

## Supplementary Materials

The following supporting information can be downloaded at: https://www.mdpi.com/article/10.3390/ijms25073894/s1.
