# Supplementary material for: Exploring the Integrated Role of miRNAs and lncRNAs in Regulating the Transcriptional Response to Amino Acids and Insulin-like Growth Factor 1 in Gilthead Sea Bream (Sparus aurata) Myoblasts
Source: Int J Mol Sci. 2024 Mar 31;25(7):3894. doi: 10.3390/ijms25073894 (PMC11011856; doi:10.3390/ijms25073894)
Supplement: Supplementary file 1 [file ijms-25-03894-s001.zip › Supplementary_Table_S1.pdf]

Table S1. Predicted interactions between lncRNAs and mRNAs significantly modified in response to treatments.

**lncRNAs ID**	**Genes**	**Correlation Index**	**Energy -ndG** (kcal/mol)
ENSSAUG00010016143	*acta1; rbm24b*	−0.86; −0.90	−0.10; −0.14
ENSSAUG00010015941	*h2az1*	−0.96	−0.10
ENSSAUG00010017089	*acta1; pin1; tcima*	−0.88; −0.81; −0.95	−0.11−0.10; −0.10
ENSSAUG00010012182	*acta1; psmb3; tcima; tnni2*	−0.88; −0.91; −0.86; −0.90	−0.15; −0.13; −0.10; −0.12;
ENSSAUG00010013622	*nupr1a; rgcc*	−0.88; −0.93	−0.11; −0.12
ENSSAUG00010015504	*nupr1a*	−0.92	−0.12
ENSSAUG00010016109	*nupr1a*	−0.85	−0.12
ENSSAUG00010017848	*igfbp6a*	−0.85	−0.14

The predicted interactions between lncRNAs and mRNAs shown are based on transcriptional correlations and bioinformatics analysis. Interactions with Pearson correlations lower than −0.80 and with predicted interaction energies lower than −0.10 kcal/mol are shown.
